# Sensitivity analysis of Repast computational ecology models with R/Repast

**DOI:** 10.1002/ece3.2580

**Published:** 2016-11-21

**Authors:** Antonio Prestes García, Alfonso Rodríguez‐Patón

**Affiliations:** ^1^ Departamento de Inteligencia ArtificialUniversidad Politécnica de MadridBoadilla del MonteMadridSpain

**Keywords:** computational ecology, individual‐based modeling, Repast, sensitivity analysis, systems biology

## Abstract

Computational ecology is an emerging interdisciplinary discipline founded mainly on modeling and simulation methods for studying ecological systems. Among the existing modeling formalisms, the individual‐based modeling is particularly well suited for capturing the complex temporal and spatial dynamics as well as the nonlinearities arising in ecosystems, communities, or populations due to individual variability. In addition, being a bottom‐up approach, it is useful for providing new insights on the local mechanisms which are generating some observed global dynamics. Of course, no conclusions about model results could be taken seriously if they are based on a single model execution and they are not analyzed carefully. Therefore, a sound methodology should always be used for underpinning the interpretation of model results. The sensitivity analysis is a methodology for quantitatively assessing the effect of input uncertainty in the simulation output which should be incorporated compulsorily to every work based on in‐silico experimental setup. In this article, we present R/Repast a GNU R package for running and analyzing Repast Simphony models accompanied by two worked examples on how to perform global sensitivity analysis and how to interpret the results.

## 1 Introduction

The computational ecology is a relatively young field which relies extensively on mathematical computational methods and models for studying ecological and evolutionary processes. It is based on the construction of predictive and explanatory models as well as the quantitative description and analysis of ecological data (Helly, Case, Davis, Levin, & Michener, 1995; Petrovskii et al., 2012). The continuous growth of computational power available for and end users, the existence of tools, and the constant increment of empirical data available makes viable for many scientists to develop and simulate tremendously complex models from their desktops. In addition, the intrinsic characteristics of ecological processes, maxim their temporal and spatial scale (Dieckmann, Law, & Metz, 2000), converts the task of carrying out controlled experiments a physical impossibility. Hence, in most cases, the only feasible alternative is to simulate the process in order to make experiments spanning the full length of ecological and evolutionary scales. The computational ecology has its roots from the successful results achieved from mathematical ecology which has proven to be an essential tool for understanding the complexities which arise from ecological interactions.

It is widely accepted that simple models with a small number of state variables and parameters provide best generalizations than the complex ones (Evans et al., 2013; Smith, 1974) with a clear distinction between simulation models and theories as separate entities handling different kind of problems. It has been recently questioned the correctness of the idea the simple models lead to generality in ecology (Evans et al., 2013). We believe that the parsimony principle must always be taken into account when developing models, but this has a different meaning depending on the modeling formalism we are using. Simplicity does not have the same meaning when the referred modeling formalism is a deterministic ordinary differential equation (ODE) or when it is applied to agent‐based modeling, as long as every modeling techniques has its own idiosyncrasy and constraints. The agent‐based modeling is a flexible and versatile abstraction where the whole system under study is described or formalized by its component units, which facilitates a more natural description of a system and the comprehension of individual properties leading to the emergent phenomena (Bonabeau, 2002).

The agent‐based models (AbM) are much more fine‐grained than their whole‐population aggregated counterpart, and as a consequence, they tend to be more complex requiring more equations, parameters, and processes in order to represent the same phenomenon. That is, not intrinsically a problem or a quality but simply a constraint imposed by the modeling formalism in use, and it is up to the modelers to find the correct trade‐off between the purpose of the model and the level of details which should be part of the model structure.

The AbM is being established progressively as a mainstream and valuable tool for modeling complex adaptive systems in many distinct areas of knowledge, ranging from social science, economics to any flavor of computational and systems science such as biology, ecology, and so on (Grimm & Railsback, 2005). The reason is, among other things, the relative ease with which detailed structural information can be incorporated into a model without the constraints of other methodologies (Hellweger & Bucci, 2009). Nonetheless, the possibility of incorporating many details comes with the cost of models with a high‐complexity level, containing many rules and parameters for which the exact values are, in many cases, hard or impossible to determine experimentally, that is what is known as parameter uncertainty. When used in the context of ecological systems, the agent‐based modeling is also known as individual‐based modeling (IbM; Grimm & Railsback, 2005).

The distinctive aspect defining what is an IbM is that individuals are represented by discrete entities and they also have a property or state variable which are unique in the population being simulated (Berec, 2002). Hence, IbM is a valuable abstraction for simulating populations, communities, or ecosystems capturing the individual variability, randomness, and their complex dynamics. It is a bottom‐up approach where the system under study is modeled using mechanistic explanations on the interacting system parts (Ferrer, Prats, & López, 2008). Therefore, the global behavior shown by the system as a whole is an emergent property derived from the local rules defining the individuals, which is particularly useful for testing different hypothesis or phenomenological explanations for the individual processes in order to verify which of them are producing the global observed behavior (Pascual, 2005). Moreover, differently from aggregate models, it is customary that IBM have a large number of state variables and parameters which in most cases are hard or directly impossible to elucidate experimentally leading to many levels of uncertainty in this kind of models. In order to tackle with the uncertainty and for making robust predictions, we have to use a sound methodology for applying what‐if analysis to check how stable are the model outputs when varying the input parameters (Thiele, Kurth, & Grimm, 2014). There exist a large set of mathematical tools for analyzing the model output which are known generically as sensitivity analysis. Normally, applying these techniques are cumbersome, requiring much effort from modelers, hindering the throughout analysis of computational models.

According to Thiele et al. 2014 most of the individual‐based models published, it tends to omit the systematic analysis of model output, mainly because modelers normally do not have the specific knowledge to implement the required methods. Therefore, it seems to be clear that the availability of simple and user‐friendly tools for experiment design and analysis would greatly help modelers to improve the formal quality of their models.

In other scientific fields, which are strongly rooted on an extensive experimentalism, it is practically impossible to conduct any kind of research without a well‐designed experimental setup and a further statistical analysis and hypothesis test. Perhaps the reasons are that these experimental fields already have a complete and mature toolbox for design and evaluation of experiments (Little & Hills, 1978; Myers & Well, 1991), leaving no room for deviation from these standards. On the other hand, silico‐based experiments are still on early stage and verification and validation procedures are not well established yet. In addition, the real value of a computational model depends much on the ability of other researchers to reproduce and enhance the results elsewhere; in other words, results must be reproducible. Hence, in order to achieve reproducibility, research methods should be stated clearly and should preferentially being backed by standard methods and software tools.

Bearing this in mind, we introduce R/Repast a GNU R package for running Repast (North et al., 2013) models from GNU R environment as well as for carrying out global sensitivity analysis on the model results. In the following sections, we will contextualize the problem providing a basic background for understanding what is being addressed in this study and we will also provide a basic description about the package functionalities. Finally, we will show three worked examples on how the package can help modelers to make the conclusions drawn from model results much more robust. The first example explores the basic aspects of bacterial conjugation process. The second is an individual‐based implementation of the classic predator–prey model enclosed as part of the standard Repast Simphony distribution. Finally, the last example was developed ex professo for this study and it is an instance of common pool problem in the context of two plasmids “sharing” the genes required for the expression of conjugative system.

## 2 Background

### 2.1 Model development

**Figure 1:**
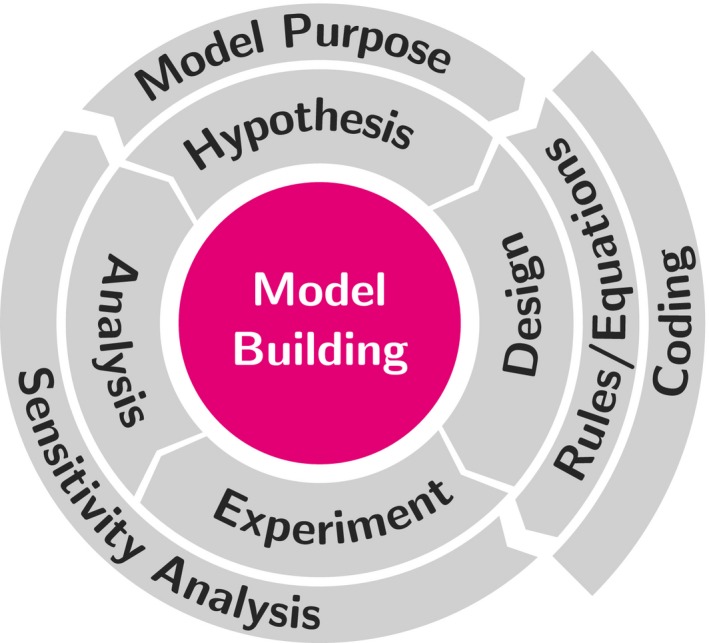
The iterative model development life cycle. This figure shows the relationship between the modeling phases and their associated tasks when applied to an individual‐based model

Model development is an iterative and objective‐driven activity, and the first step required to develop a model is having a clear and ideally unambiguous statement about the model purpose. Therefore, every experimental study carried out using modeling and simulation should follow the experimental life cycle based on the successive sequence of four cyclic steps, starting from (1) conjecture, which defines the model purpose and why the model is being developed; (2) design phase, where the model is translated to some runnable implementation; (3) experiment step, which means the execution of model following a well‐established plan oriented to confirm or reject the initial conjecture; and finally, the (4) analysis step, where the data generated in the previous step is analyzed with a sound methodology which will generate new insights, uncover model flaws, and iteratively improve the initial conjecture and design (Box & Draper, 1987). A simple graphical representation of these four iterative steps is shown in Figure 1.

Part of design phase consist in converting the model equations and rules to a computer code implementation. Currently, there are several frameworks available for developing individual‐based models. These frameworks are designed to address some specific requirement such as usability (Tisue & Wilensky, 2004), flexibility or scalability (Luke, Cioffi‐Revilla, Panait, Sullivan, & Balan, 2005; North et al., 2013), or support to multiple paradigm, such as AnyLogic (Emrich, Suslov, & Judex, 2007). Certainly, the most widespread framework in ecological modeling is NetLogo (Tisue & Wilensky, 2004) which is considered to provide an easier development environment based on extensions to Logo paradigm especially suited for those which are not much familiar with modern programming languages. One of the main drawbacks of NetLogo is the scalability. NetLogo tends to show some performance issues when simulating a large number of agents. On the other hand, Repast Symphony framework has a steep learning curve but provides a fast and flexible java‐based environment with many interesting features for simulating large‐scale computational ecology models. These features include, among others things, the integration with Weka, exporting the model output to R environment, support for running distributed batch simulations, and some built‐in facilities for parameter sweeping (North et al., 2013). Finally, Mason is, in some extent, very similar to Repast but less mature than it is; it has been designed focusing on providing faster execution speeds (Luke et al., 2005). Of these frameworks, only AnyLogic provides integrated sensitivity analysis capabilities, whereas the other frameworks NetLogo, Repast, and Mason, which are all free software, do not have built‐in support to sensitivity analysis.

The Repast framework is widely used in many different fields for building individual‐based simulation models of dynamic processes (Gutfraind et al., 2015; Tack, Logist, Noriega Fernández, & Van Impe, 2015; Watkins, Noble, Foster, Harmsen, & Doncaster, 2015). In addition, Repast also has a framework for high‐performance computing using the C++ programing language with similar conceptual entities as those found in Repast—java. Repast also has support for running GNU R code (Crawley, 2007; R Core Team, 2015) from inside the user interface, but until now, it has not been feasible to run Repast models from R environment for controlling model in order to implement experimental designs, calibration, parameter estimation, and sensitivity analysis, therefore hindering a throughout and comprehensive validation of individual‐based models developed using Repast Simphony.

### 2.2 Sensitivity analysis

Because of sensitivity analysis is a broad and complex subject, a throughout discussion would be lengthy and out of the scope of this work. Instead, we will try to provide a more amenable and practical approach keeping the discussion at a general level but rigorous enough to let the practitioners gain the knowledge required to understand, apply, and interpret the results. For a more detailed review, please refer to Saltelli, Tarantola, Campolongo, and Ratto 2004 and Pianosi et al. 2016. It is interesting to start the discussion providing the exact meaning of some the many expressions which are used commonly in the analysis of models. There are several terms used in the context of sensitivity analysis for which is important to provide the formal meaning. For instance, the jargon of sensitivity analysis includes model calibration and parameter estimation which many times are used as they were equivalent, even though they are different objectives. Other terms such as uncertainty analysis, omitted variable bias, objective function, or cost function are also important part of SA lexicon.

Generally speaking, the objective of SA is to understand the effect of varying input factors on the model output (Saltelli et al., 2004). Under this very general statement, we have a wide range of methods and techniques which are suitable for distinct kinds of models. In order to improve this definition, it is convenient to provide a more formal definition to the entity which is the target of SA: the model. Formally speaking, a model is a functional relation between a number *k* of input factor, also called independent or predictor variable and the output variable, sometimes referred as dependent or response variable (Box & Draper, 1987) as depicted by the expression $\eta \;=\;f(x_{1},\;x_{2},\;\ldots ,\;x_{k})$, being η is the average value of response variable considering any specific setting for the input factors $x_{i}$. Therefore, the value of a single model run is given by $y\;=\;f(x_{1},\;x_{2},\;\ldots ,\;x_{k})\;+\;\epsilon$, where ε is difference between the value of *y* and the expected value *E*(*y*) = η. The error ε is consequence of stochasticity introduced by design in the structure of model to capture the population variability. Finally, recognizing that most real‐world models usually have more than one response variable, the structure of an individual‐based model *M* can be generalized for *n* outputs as can be seen below

$$M=\left\{\begin{array}{c} y_{1}=f_{1}\left(x_{1},x_{2},\ldots ,x_{k}\right)+\epsilon \\ y_{2}=f_{2}\left(x_{1},x_{2},\ldots ,x_{k}\right)+\epsilon \\ \vdots \\ y_{n}=f_{n}\left(x_{1},x_{2},\ldots ,x_{k}\right)+\epsilon \end{array}\right.$$

Therefore, being $y_{i}$ some output of model *M*, the model calibration process consists in comparing these outputs to some reference values (Zeigler, Praehofer, & Kim, 2000) which are normally, in the case of ecological or biological studies, experimental or observed data. The target of calibration process is minimizing the discrepancies between simulated and reference values. The function used for computing how far $y_{i}$ output is from the reference values is known as objective function or cost function. There are many options for implementing the objective function and the only requirement is that the return of objective function should be inversely proportional to the quality of fit, being zero the return value for the perfect fit. Common implementations for objective function are based on the definition of acceptable ranges, least squares, or even a combination of both. For instance, let $y_{i}$ be the output of some hypothetical model *M*, assuming this variable represents the net reproductive rate $R_{0}$. The reference values $R_{v}$ for the output variable must fall between 0.8 and 1.2; hence, any $y_{i}$ value within this interval is considered to have a perfect fit, bearing this in mind the cost function could be given by the following expression

$$C\left(y_{i}\right)=\left\{\begin{array}{cc} 0, & \text{if}\;0.8\;\leq \;y_{i}\;\leq \;1.2\\ 1, & \text{otherwise} \end{array}\right.$$

That is, what is known as categorical calibration criteria (Thiele et al., 2014). The main drawback of this approach is that it does not provide any information about how far is the response value from the reference value. A better alternative is to apply some distance function $d(y_{i},\;R_{y})$ to the output and the reference values, even standalone or in combination with categorical calibration. The most commonly used metric is some of the multiple forms of squared deviation, but any distance function can be alternatively employed as long as two properties hold: $d(y_{i},\;R_{y})\;=\;0$ if $x_{i}$ and $R_{y}$ are equal and $d(y_{i},\;R_{y})\;>\;0$ when $x_{i}$ and $R_{y}$ are not equal.

While calibration is a general term, meaning fundamentally the comparison of some value to a reference value, the term parameter estimation has a more subtle and specific goal. The parameter estimation is normally considered an inverse problem because the objective is finding the values for the model parameters providing the best adjustment to the reference values. In other words, knowing the expected values for response variable, the target is estimating the suitable values for the model parameters. Usually, the terminology parameter refers to the constants which are part of models with clear distinction between parameters and independent variables (Beck & Arnold, 1977), for instance, in the growth differential equation shown below

$$\frac{dN}{dt}=rN,$$

the model parameter would be only the growth rate *r* and the independent variable the time, but for the purpose of this study, we consider indistinctly the model constants and independent variables as being parameters.

The two main objectives of sensitivity analysis are understanding how robust are the model results considering the existing uncertainties and quantifying the effect of input factors on the variance of output (Law, 2005; Pianosi et al., 2016; Saltelli et al., 2004). The intrinsic characteristics of individual‐based models which relies on mechanistic descriptions favors the production of models with many subprocesses, state variable, and parameters. The design is normally based on incomplete knowledge resulting in several levels of uncertainties in the model parameters, in the model response variables, and in the model structure itself. The model structure is also related to the identifiability problem where not all model parameters can be uniquely estimated. The sensitivity analysis can be also used to assess the effect of model structure on the output considering the alternative model implementations as being another parameter. This can be useful for analyzing the omitted variable bias, which basically means that some parameter of model can be over or underestimated because another important parameter was not included in the model structure. The sensitivity analysis can be carried out letting the parameters varying over the full range of parameter space or restricted to a small region close to the average value, respectively, referred as global sensitivity analysis and local sensitivity analysis. Sensitivity analysis can also be performed varying one factor at a time (OAT) leaving all others fixed or varying all factors at the same time (AAT). The application of second method is required in order to capture interaction between parameters and nonlinear effects.

The central point of SA methodology is the estimation of sensitivity indices or coefficients. The sensitivity coefficients allow the quantitative comparison of the contributions from distinct parameters to the model output. In its classical form (Beck & Arnold, 1977), the sensitivity indices are defined as the first derivative with respect to some model parameter $x_{i}$. Considering the general model *y* = *f*(*X*), being *X* the parameter vector of size *k*, the sensitivity index $S_{i}$ is given by

$$S_{i}=\frac{\partial Y}{\partial x_{i}}$$

It is also important to take into account that the partial derivatives can have different units, hence can be necessary to scale them in order to make them comparable. In this approach, input factors are perturbed one‐at‐a‐time, being that measure of sensitivity suitable for local SA (Pianosi et al., 2016).

**Figure 2:**
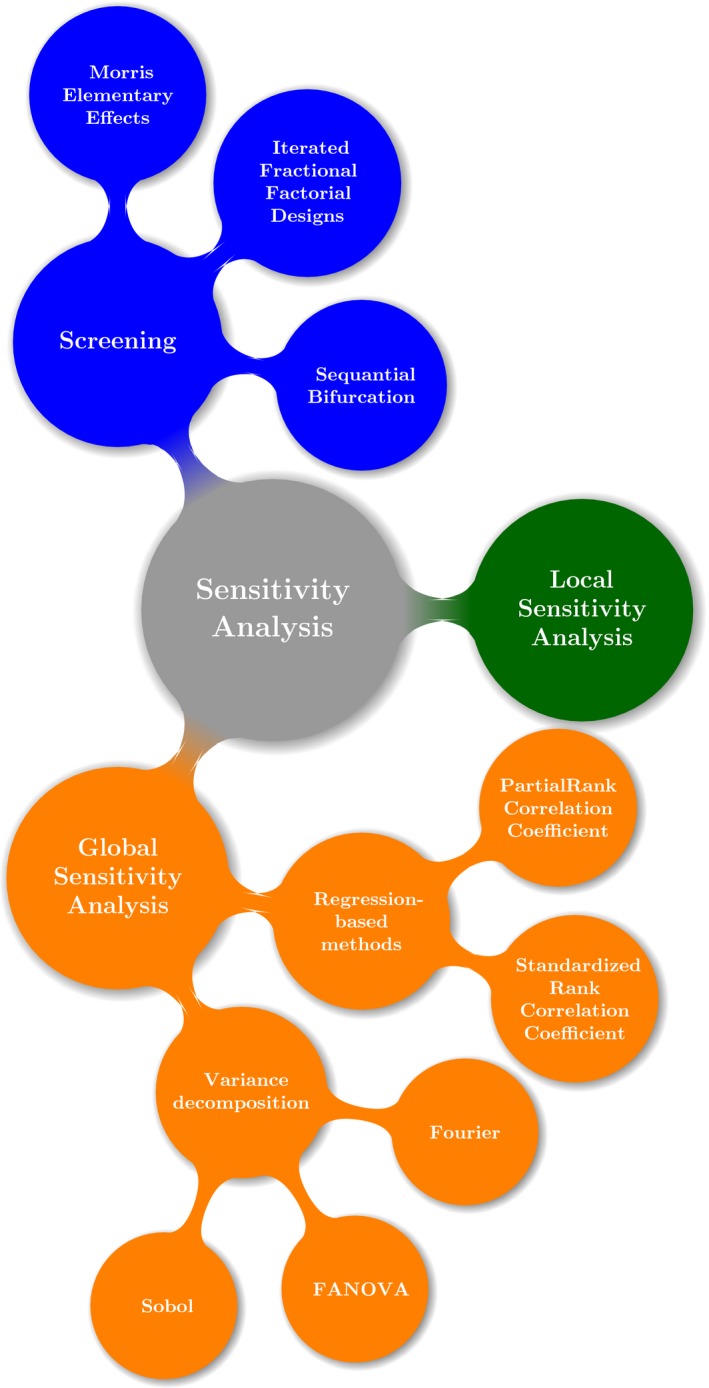
The different types of sensitivity analysis and their associated methodologies and techniques

Several methods to estimate sensitivity indices which are adequate for global sensitivity analysis are available, such as metamodeling approach (Happe, Kellermann, & Balmann, 2006), correlation‐based methods, regression‐based methods, Fourier amplitude sensitivity test (FAST; Xu & Gertner, 2011), for a more in‐depth discussion, please refer to Thiele et al. 2014, Saltelli et al. 2004, Saltelli 2008, Pianosi et al. 2016 and Pujol et al. 2015. The Figure 2 shows how the different methods for assessing the importance of input factors in simulation models are related, also including screening techniques (Saltelli, Andres, & Homma, T. 1995; Bettonvil & Kleijnen, 1996). In this study, we will focus on those methods based on the variance decomposition which are suitable for a wide range of situations, including those which are commonly found in individual‐based models, such as nonlinear mappings between input factors and outputs variables (Zhang & Rundell, 2006). In addition to first‐order effects, the variance decomposition methods also allow the quantification of second‐order effects sometimes referred as total‐order effects. Total‐order effect indices are useful for the assessment of the interaction between factors which cannot be expressed by a simple linear superposition.

One of main drawbacks for applying variance decomposition methods on large spatially explicit individual‐based models is the requirement of very high number of model evaluations in order to produce consistent results (Herman, Kollat, Reed, & Wagener, 2013). An alternative approach, in those cases where it is impractical or computationally unfeasible a fully quantitative analysis, is the application of the Morris screening method. The Morris method delivers qualitative information allowing to rank the importance of input factors requiring lees model evaluations, which in some case can one order of magnitude be inferior to the Sobol method (Saltelli, 2008).

The Sobol is a method for sensitivity analysis based on the decomposition of the variance of model output and is particularly suitable for discovering the effect of high‐order interactions between input factors. The interaction means nonlinearity where the total effect of two input factors $x_{1}$ and $x_{2}$ on the model output *Y* are not equivalent to the sum of the individual effects. The general form of sensitivity indices for Sobol methods is shown in Equations (1) and (2), respectively, the first‐order and total‐order indices.

(1) $$S_{i}=\frac{V_{i}}{V(Y)},$$

(2) $$S_{\mathrm{Ti}}=1-\frac{V\left(Y\right)-V_{i}}{V(Y)},$$

where the terms $V_{i}$ and *V*(*Y*) are, respectively, the variance contribution attributed to the ith parameter and the total variance. The expression $V\left(Y\right)-V_{i}$ represents the total variance with exception of the variance which is generated by the parameter *i*. The total‐order index $S_{\mathrm{Ti}}$ is the contribution of all input parameters but one, the *i*th parameter, and hence estimating the effect of that parameter on the variance reduction (Saltelli, 2008).

The total variance *V*(*Y*) for a model with *n* input parameters can be expressed as shown in Equation (3) as long as the orthogonality of input factors precondition holds.

(3) $$V\left(Y\right)=\sum_{i}V_{i}+\sum_{i\;<\;j}V_{ij}+\sum_{i\;<\;j\;<\;k}V_{ijk}+\cdots +V_{12\ldots n},$$

being *V*(*Y*) the total variance from model output and the components $V_{i}$, $V_{ij}$, and $V_{ijk}$, respectively, the variance contribution from the parameter *i*, the variance contribution form input parameters *i* and *j*, and the variance contribution form input parameters *i*,* j*, and *k*. Finally, the component $V_{12\ldots n}$ expresses the interactions from all parameters present in the model.

The application of Sobol method, as have been mentioned, can be computationally expensive and sometimes could be useful to reduce the problem dimensionality filtering only the most significant parameters or even simplifying the model structure considering only the parameters accounting for the most of the variability in the model output. It can be accomplished using the Morris screening method to rank the importance of input parameters. The Morris method is an OAT method, meaning that it changes just one factor keeping all other input parameters fixed. The input factors are allowed to vary in discrete levels within the relevant parameter range (Morris, 1991). The method is considered to be more effective when the number of most significant input parameters are a small subset of model parameters (Saltelli et al., 2004).

The original work of Morris 1991 defines two metrics for ranking input factors which are depicted by μ and σ values.^1^ Further, another metric termed $\mu^{*}$ has been suggested by Campolongo, Cariboni, and Saltelli 2007 which use absolute values in order to handle effects of distinct signs canceling each other. These metrics for ranking input factors are calculated from what has been termed elementary effects. Therefore, considering a model with *k* input parameters and being $X\;=\;(x_{1},x_{2},\cdots ,x_{k})$ any value from the region of experimentation Ω, the elementary effects are calculated according to the Equation (4).

(4) $${\text{ee}}_{i}\left(X\right)\;=\;\frac{y\left(x_{1},\ldots ,x_{i-1},x_{i}+\Delta ,x_{i\;+\;1},\ldots ,x_{k}\right)-y\left(X\right)}{\Delta }.$$

The region of experimentation Ω is a grid defined by the number of *k* input factors and by the *p* discrete levels for every parameter. The recommendations for the values of *p* and Δ are, respectively, that the first should be an even number of levels and the second calculated by the expression Δ = *p*/(2(*p*−1)) (Morris, 1991; Saltelli et al., 2004). The value of Δ has important implications in the model analysis. It has been shown that in some situations, choosing an alternative value calculated as Δ = 1/(*p*−1) can detect nonmonotonic behaviors such that the suggested standard calculations are not able to capture otherwise (van Houwelingen, Boshuizen, & Capannesi, 2011).

The metrics of Morris method are calculated over the $F_{i}$ and $G_{i}$ distributions for every input parameter. These distributions are generated taking random samples of X from Ω for calculating the elementary effects, and the only difference between them is that $G_{i}$ uses the absolute values of elementary effects $|{\text{ee}}_{i}\left(X\right)|$ as described in Campolongo et al. 2007 and Saltelli 2008. The estimation of Morris metrics are carried out by taking *r* samples from $F_{i}$ and $G_{i}$ distributions according to the Equations (5), (6), (7).

(5) $$\mu =\sum_{i\;=\;1}^{r}\frac{{\text{ee}}_{i}\left(X\right)}{r},$$

(6) $$\mu^{*}=\sum_{i\;=\;1}^{r}\frac{|{\text{ee}}_{i}\left(X\right)|}{r},$$

(7) $$\sigma =\sqrt{\sum_{i\;=\;1}^{r}\frac{{\left({\text{ee}}_{i}\left(X\right)-\mu \right)}^{2}}{r}}.$$

These three metrics can be used to extract valuable information about the model behavior, in addition to ranking the input factors. For instance, a low value of μ and a high value of $\mu^{*}$, points that the input factor under scrutiny, possibly has a nonlinear behavior having different signs in function of the system trajectory (Saltelli et al., 2004). A high value of μ indicates that the input has a monotonic effect on the model output.

The sensitivity analysis methods require significant samples from input space in order to provide reliable results. It is customary to choose between some experimental design (Hicks, 1993) for generating the collection of input parameters needed by evaluating the model and allocating the variance contribution of every model parameter. The most generally applied sampling schemas are based on random sampling, full factorial designs, or Latin hypercube sampling.

## 3 Overview of R/Repast Package

In the previous sections, we had seen some fundamental ideas on model building and the role occupied by sensitivity analysis methods in the iterative modeling life cycle. We have also introduced the basic principles of sensitivity analysis focusing on two main techniques namely the Morris Elementary Effects Screening (Morris, 1991) and the Sobol GSA method for variance decomposition (Saltelli, 2008). Both methods have a wide range of applicability, making them suitable for their use in the analysis of individual‐based models. These methods require the model to be evaluated many times with a different set of input parameters, making completely impractical undertaking a manual analysis introducing individual parameters manually on a graphical user interface. The Repast is an extremely flexible framework for object‐oriented development of AbM using Java language, but it lacks model analysis tools. On the other hand, the GNU R is a superb open‐source tool for data analysis with a vast and active community developing and adding new methods to the core R system. Bearing this in mind, we introduce our package R/Repast which bring together the best of both worlds. Roughly speaking, the R/Repast package have two main objectives: (1) Provide an interface for running Repast models from R and gathering the simulation data generated and (2) automating the application of sensitivity analysis and simple model calibration methods to the Repast models. The R/Repast is an open‐source project delivered under the MIT license system. The package provides a powerful and simple R Application Programming Interface (API) which reduces the code required for running the most commonly used experimental methods suitable for. The software and the user manual can be downloaded from CRAN website and the complete project source code from GitHub repository. Both are available, respectively, from the following URLs:

- https://cran.r-project.org/web/packages/rrepast/
- https://github.com/antonio-pgarcia/RRepast

### 3.1 Design

**Figure 3:**
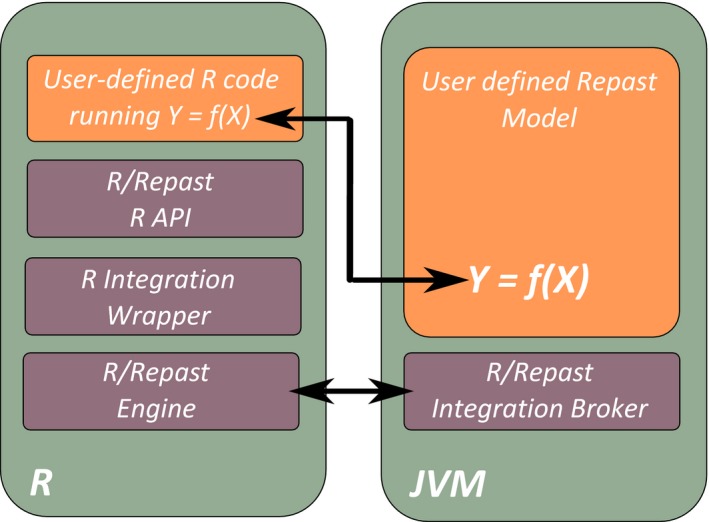
The R/Repast general architecture. The scheme shows in the left box the R environment and the associated components of R/Repast. The right box represents the Repast Simphony model running within a Java Virtual Machine as well as the R/Repast integration broker component

The R/Repast was intended primarily for invoking Repast Simphony models from inside GNU R environment. Additionally, the package contains more high‐level and value‐added features for experimental design and experiment analysis to address the specific need of individual‐based models. The underlying implementation idea is to provide a set of turnkey features for facilitating the task of applying the sensitivity analysis to models. Functionally, the package consists of four modules which interoperate together for instantiate and running the Repast code inside R. These four components are (1) the Repast Integration Broker, (2) the Repast Integration Engine, (3) The R Integration wrapper, and finally, (4) the R API for Experiment design. A schematic view of package architecture is shown in Figure 3.

The R/Repast integration broker and the R/Repast engine are both written in java code and are required for instantiating and loading the Repast Simphony model in batch mode. The R/Repast engine contains also the required hooks for transferring the model output data from Java to R environment. The engine can transfer data from aggregated dataset defined by the modeler on the Repast model. An aggregated dataset is a Repast Simphony entity used to collect data about the simulation model agents which can be used for plotting or saving the model output data to a file using a file sink. A *File Sink* is Repast component for saving simulation data to a file. The aggregated datasets use some kind of aggregate operation, such as counting, averaging, summing, or any other used defined aggregate operation (North et al., 2013; North et al., 2005). The R integration wrapper is the R code for linking together the R and Repast subsystems. This module consist of several wrapping functions for encapsulating the Java code calls implemented using the *rJava* package (Urbanek, 2016). These functions are prefixed with the [Engine] keyword and, although exported in the R/Repast package, they are not intended for general use.

### 3.2 The R/Repast R API

The module entitled R/Repast R API is the primary entry point for the user‐defined code and relies on the subsystems mentioned previously for providing three group of functionalities for facilitating modelers to analyze the simulation output. These group functionalities are the following:

- Execution and control of Repast Simphony code.
- Basic functions for experimental design.
- High‐level functions for a complete experiment in one call.

The functionalities on the first group are those required for the basic interface between Repast and R system, such as instantiating and running a Repast Simphony model, retrieving the declared model parameters, getting their default values, setting parameter values as well as running basic experimental designs and saving simulation data. The list of these functions are shown in Table 1.

**Table 1:** The basic R/Repast Application Programming Interface functions. These functions are used for loading and modifying the default parameters defined for model and also for running the simulation

Function name	Description
Model (*d*,* t*,* o*,* l*)	This function creates an object instance for linking the Repast model to an R object. The required parameters are the directory where the model has been installed *(d)*, the duration of simulation in Repast ticks *(t)*, the name of any aggregated dataset of model for draining data generated by the model simulation *(o)*, and a Boolean flag *(l)* which tells the function to call the Load method. The default value is FALSE
Load (*m*)	This function loads the Repast scenario from model's directory. The only required parameter *(m)* is an instance of Repast Model created with previous function
Run (*m*,* r*,* s*)	The purpose of this function is to execute a single round of simulation using just one parameter set. The parameters for this function are a model instance *(m)*, the number of repetitions *(r)*, and a collection of random seeds *(s)* to be used for each one of the repetitions. The only required parameter is the model instance, created with the *Model()* function. The default value for *r* is one
RunExperiment (*m*,* r*,* d*,* F*)	Execute a complete experimental setup for different sets of parameters. The parameters required are a model instance *(m)*, the number of replications *(r)*, the experimental design *(d)*, and finally a user‐provided calibration function *(F)*. The experimental design parameter is an R data frame containing a complete set of model's parameter per row. The function returns a list with three data frame elements: the *paramset*, the *output*, and *dataset* which holds, respectively, all simulated input parameters, the result of user provide calibration function, and the complete dataset produced during the experiment execution
GetSimulationParameters (*m*)	Returns the complete list of parameters declared by the model. The parameter *(m)* is an instance of Repast model generated with *Model()* call described previously
SetSimulationParameters (*m*, *p*)	Modify several parameters at once
SaveSimulationData (*t*,* e*)	Exports the results of Run or RunExperiment to a csv or excel files. The parameters *t* and *e* are, respectively, the format of exported data (xls or csv) and the experiment results returned by *RunExperiment*()

The second group of methods within R/Repast R API contains the functionalities required for setting up and applying a complete experimental design to a Repast simulation model. The group include functions for adding the input factors and the relevant input range which the modeler wants to evaluate. The group also have functions for generating the experiment inputs using different sampling approaches. It is not required to add as input factors all declared model parameters, the modeler can just evaluate a small subset keeping the other factors fixed. The functions of this group are presented in Table 2.

**Table 2:** The experimental setup Application Programming Interface functions. These functions are used for experimental design, parameter calibration, and sensitivity analysis

Function name	Description
AddFactor (*f*,* l*,* k*,* b*,* u*)	Creates the parameter collection for the experimental setup. The function requires the data frame *(f)* where parameter will be added, if this parameter is not provided, a new data frame will be created. The second parameter *(l)* is the random function used internally, the default value is *runif* which will be the valid choice in many cases, the next parameter *(k)* is the name of factor, the value provided must match some parameter defined in the repast model. The following two parameters *(b)*,* (u)* are the lower and the upper range, respectively. The function returns the updated *(f)* data frame with the new parameter
AoE.RandomSampling (*n*,* f*)	Also known as Monte Carlo sampling, generate an experimental design based on making random samplings of parameter space. The function takes two parameters, the sample size *(n)* and the factor *(f)* data frame created using AddFactor(). The function returns the design matrix for the provided parameters
AoE.LatinHypercube (*n*,* f*)	Generates an experimental design using the Latin Hypercube stratified sampling technique which is a more efficient sampling scheme, in terms of model evaluations, than the pure random sampling. The parameters *(n, f)* and return values are the same already described for the function AoE.RandomSampling()
AoE.FullFactorial (*n*,* f*)	Creates a factorial design where the effects of all independent variables of model are studied simultaneously, which implies many more model evaluations. The parameters *(n, f)* and return values are the same already described for the function AoE.RandomSampling()
BuildParameterSet (*d*,* p*)	Constructs the data frame required for executing RunExperiment(). The function takes two parameters: the design matrix *(d)* created with one of previous functions and the declared parameters *(p)* defined in the Repast Model with the default values retrieved using the function GetSimulationParameters(). The functions return a data frame with varying and fixed parameters for the experimental setup of choice

Finally, the third group contains the “Easy” API functions. These functions are intended to provide a complete method implementation which is accessible using just one R function call. The user has to provide the directory location where the Repast model is installed, the objective function, and the parameters relevant to the specific method. The currently available Easy API methods are presented in Table 3. The objective function is a user‐defined R function over the model output for calculating and returning a cost metric for the simulation outputs of interest. The return of objective functions is the target for the application of the analysis method.

**Table 3:** The easy Application Programming Interface functions. These functions are the preferred entry point for the eventual users. These “Easy” functions lump together a complete experiment task in just one call, reducing the number of lines of code required

Function name	Description
Easy.Stability (*d*,* o*,* t*,* f*,* s*,* r*,* v*,* F*)	Evaluate the behavior of model output in order to determine the minimum required number of replication of the chosen experimental setup. The function accept the following parameters: the model installation directory *(d)*, the aggregated data source defined within the Repast model *(o)*, the simulation time in Repast ticks *(t)* which default value is 300 ticks, the input factors to be sampled *(f)* created with the previously mentioned function AddFactor(), the number of parameter samples *(s)*, the desired number of replications to be tried *(r)* being the default value 100, the output variables of interest which will be checked for their stability and convergence of the coefficient of variation *(v)*; if this parameter is left empty, all output variables are checked and finally the user provided calibration function *(F)* for determining the best input parameter combination
Easy.Morris (*d*,* o*,* t*,* f*,* p*,* s*,* r*,* F*)	This function performs all required tasks for carrying out the method of Morris for screening. The parameters are practically the same as described for the previous function with exception of parameters *(p)* and *(s)* which are, respectively, the levels of input factors and the number of sampling points of Morris method (Pujol et al., 2015)
Easy.Sobol (*d*,* o*,* t*,* f*,* n*,* r*,* F*)	Encapsulate all required steps for performing sensitivity analysis using Sobol method. The method of Sobol is a global sensitivity analysis technique based on the decomposition of output variance (Pujol et al., 2015; Saltelli et al., 2004). The parameter semantics are the same already described: the model installation directory *(d)*, the aggregated data source defined within the Repast model *(o)*, the simulation time in Repast ticks *(t)* , the input factors to be sampled *(f)*, the sample size *(n)*, the desired number of replications *(r)*, and calibration function *(F)*
Easy.Calibration (*d*,* o*,* t*,* f*,* n*,* r*,* F*)	This function estimates the best set of input parameters or factors, performing a set of model executions in order to sample the calibration function. The objective of this function is to minimize the output of calibration function provided by the user
Easy.Setup (*d*,* l*)	The parameters *(d)* and *(l)* are, respectively, the directory where repast model is installed and the location of R/Repast deployment directory. If omitted, it assumes as the default value, the directory where the Repast model is installed. The function is required for automatically making the changes in the model configuration for adding the integration code, for deploying the Java *jar* files with the integration code, and for preparing the deployment directory. That directory will hold the JVM logs and the saved model output datasets

### 3.3 The objective function interface

The last piece of R/Repast architecture is the definition of the objective function which actually allows the flexible definition of the model analysis target decoupling it from the Repast dataset output. As we have mentioned previously, any model is a functional relationship between a vector of input parameters *X* and a scalar dependent variable *y* and expressed as *y* = *f*(*X*). On the other hand, usually the dataset collected from Repast model execution will be a time series where the aggregated measure will be collected at fixed intervals. Therefore, some transformation must be applied in order to obtain a value consistent with the functional definition. In addition, even though the value returned from the Repast model was a scalar one, it would add much more maintainable and flexible to act upon it directly from R without making changes in the Repast code. The objective function is also necessary for calibrating, where the output values are compared to some reference data or even for more complex tasks, such as tuning oscillations in the population output. It is also the place for normalizing he model outputs. The objective function is a required parameter for all methods presented here.

**Figure 4:**
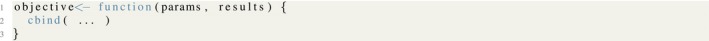
The skeleton of objective function. The function has two parameters and must return a one or more scalar values

The specification of R/Repast requires the objective function having two input parameters. The first input parameter for the objective function is the input parameter set used for executing the Repast model, the second parameter is the results generated by executing the model and corresponding to and aggregated dataset in the Repast model. The objective function must return one or more scalar values grouped using the *cbind*() (Crawley, 2007) R function. The complete function signature is shown in Figure 4.

## 4 Examples Overview

In the next sections, we will provide examples on how the R/Repast can help modelers on the analysis of their simulation models. Three examples will be used for illustrating the application of some package's functionalities and what kind of information these functions can offer about the simulation outputs. For clarifying what every model does, a summary version of Overview, Design concepts, and Detail (ODD) will be given for facilitating a general idea about these models. The ODD is a protocol (Grimm et al., 2006, 2010) which has been proposed as a standard way to specify and describe individual‐based models. A brief description on the model structure and parameters will be given in order to allow the readers to understand the kind of questions the model is intended to answer and how R/Repast can be used for analyzing the model outputs. The last section for each model under the title of *Model analysis* is not part of ODD protocol, but it is included to show the results of running the R/Repast model analysis methods.

The first model used as example here is a spatially explicit individual‐based representation of bacterial conjugation using BactoSIM for simulating the plasmid spread on a surface‐attached bacterial colony (Prestes García & Rodríguez‐Patón, 2015a, b). The example will be used for showing the application of *Easy.Stability* method for finding the number of replications of simulation experiments required for obtaining consistent outputs. The second example is a Repast implementation of the omnipresent predator–prey model describing the interaction between two species. This one is part of examples coming along the standard Repast distribution and will be used for showing the application of *Easy.Morris* function. Finally, the third example is an instance of the common pool problem in the context of bacterial conjugation. This model was developed exclusively for this study. This model will be used for exemplifying the use of *Easy.Sobol* method. The complete sources for all projects are available, respectively, in the following locations:

- BactoSIM: https://github.com/antonio-pgarcia/haldane
- Predator‐Prey: The sources come with the Repast distribution.
- T4SS Common Pool: https://github.com/antonio-pgarcia/PoolT4SS

For convenience, in order to facilitate the experiments shown in this article being reproduced elsewhere, we also provide the prebuilt installers for the three projects mentioned previously. The installers can be downloaded from URL shown below:

- BactoSIM: http://goo.gl/YYIt1o
- Predator‐Prey: http://goo.gl/cJ5z2r
- T4SS Common Pool: http://goo.gl/zq4LH0

In order to reproduce the examples shown in the next sections, it is required a computer with a Java JVM and GNU R installed. The examples have been produced and tested on a windows box with java 1.8 and GNU R 3.3.1. If these preconditions are met, just proceed to download and install the examples and the R/Repast package. The installation of R/Repast is carried out using the install command install.packages (“rrepast”) on the R environment. Once the previous steps have been completed, just copy and paste the examples shown in this article, taking care of changing the references to the model installation directory to the directories where the models have been installed locally.

## 5 Example 1: BactoSIM

Normally, one of the advantages of using individual‐based models for biological or ecological processes is the possibility of incorporating variability at an individual level. Therefore, unlike deterministic model, in order to get trustworthy results, the simulation must be repeated a number *N* of times to achieve stable value on the output variance. The objective of the first example is to show the application of a simple method for finding the minimal number of replications of a simulation model which is required for the variance of response variables become stable, converging to a common value. A straightforward way to determine the output stability has been suggested in Thiele et al. 2014 and Lorscheid, Heine, and Meyer 2012 and consists in to compute the coefficient of variation^2^ of the output of interest with and increasing number of repetitions while keeping the input parameters fixed. The number of replications for which the values of coefficient of variation stop to vary is the minimum number of repetitions necessary for getting robust results. In R/Repast, we have implemented that method which is accessible through the *Easy.Stability* API call.

For this example, the BactoSIM (Prestes García & Rodríguez‐Patón, 2015a, b) model will be used. This is an individual‐based model of bacterial conjugation process. The bacterial conjugation is a form of lateral genetic transfer which occur naturally in bacterial colonies (Arutyunov & Frost, 2013). The conjugation consists in the transference of a conjugative plasmid from a donor cell to a recipient cell. The plasmids are small circular DNA sequences which replicates independently from the main chromosome of their hosts (Bergstrom, Lipsitch, & Levin, 2000). The conjugation is considered one the causes of the rapid evolution and adaptation of bacterial colonies and the spread of antibiotic resistance (Chen, Christie, & Dubnau, 2005; Slater, Bailey, Tett, & Turner, 2008). The BactoSIM model is currently being used for an evaluation of the main factors governing the plasmid dispersion. A preliminary evaluation has shown that the point in the cell cycle is the principal factor responsible for the global dynamics of plasmid infective dispersion (Prestes García & Rodríguez‐Patón, 2015a, b) which is consistent with some observations (Seoane et al., 2011) taken from individual bacterial cells.

### 5.1 Model description

The model description follows the ODD protocol for describing individual‐based models (Grimm et al., 2006, 2010). The model is implemented in java language using Repast Simphony agent‐based simulation framework (North et al., 2013).

#### 5.1.1 Purpose

The objective of this model is the assessment of the best strategy for modeling and implementing the conjugation rule which provides the best fit to experimental data and better captures the most plausible process structure.

#### 5.1.2 Entities, state variables, and scales

The model comprises two entity types, namely the bacterial individuals or agents and environment. The environment contains the rate limiting number of nutrient particles required for the cell metabolism and growth. All agents evolve in a computational domain defined by a 1000 × 1000 μm squared lattice divided in $10^{6}$ cells of 1 × 1 μm representing a real surface of 1 mm${}^{2}$. In this model, the *agents* representing bacterial cells are defined individually by two main state variables, namely the *plasmid infection state* and the $t_{0}$. The plasmid infection states are Q=R,D,T and the respective transition function for conjugative plasmids, δ is shown in Equation (8). For the *oriT* construction only, the first transition rule applies as transconjugant cells are sterile. The $t_{0}$ is the time of cell birth or the time of the last cellular division, and it is employed in the estimation of agent doubling time used in the division decision rule. The T4SS pili is also taken into account and the agents have a state variable representing the number of pilus already expressed and available in cell surface.

(8) $$\delta =\left\{\begin{array}{c} \left(D,\;R)\to (D,\;T\right)\\ \left(T,\;R)\to (T,\;T\right) \end{array}\right.$$

Finally, the environment will hold the initial nutrient concentration for every lattice cell. In the model initialization, a fixed amount of substrate particles will be distributed evenly over all lattice sites.

#### 5.1.3 Process overview and scheduling

**Figure 5:**
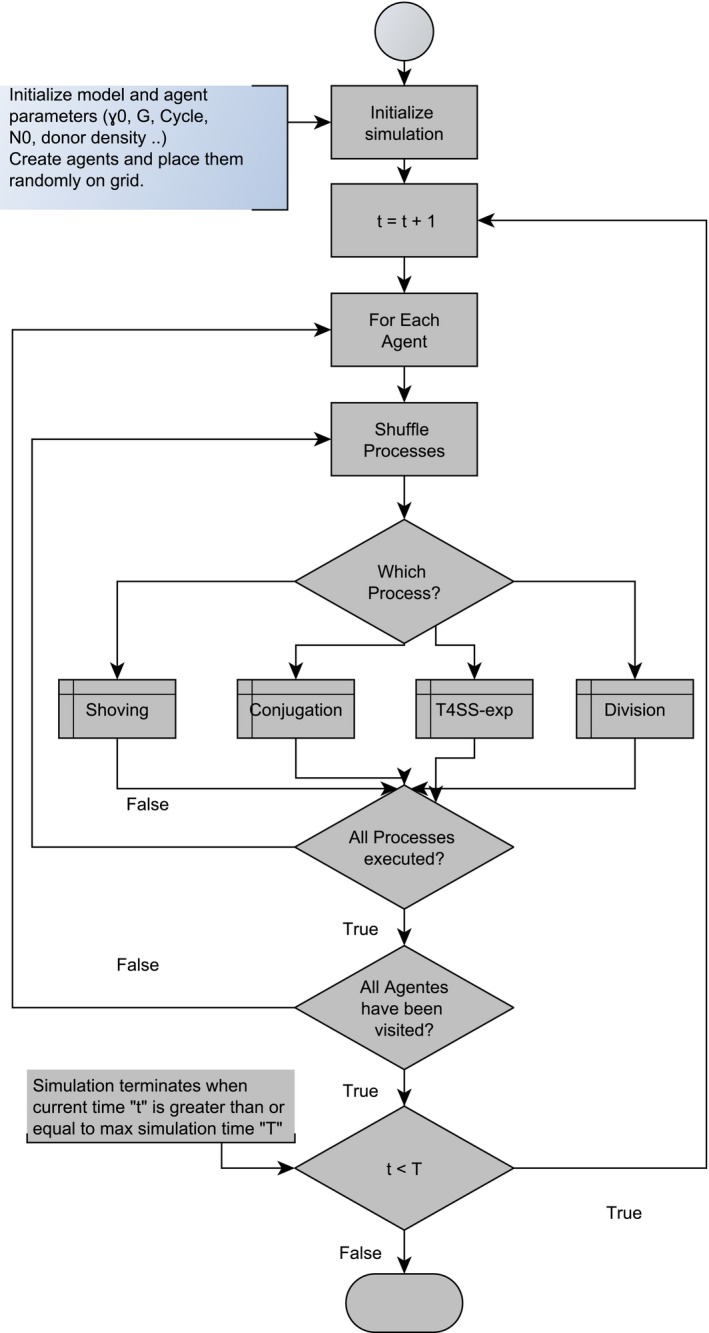
The flow diagram showing the overview on how bacterial process are scheduled in the BactoSIM simulation model

The dynamics of bacterial conjugation is modeled as the execution of following set of cellular processes: the cellular division, the T4SS pili expression, the shoving relaxing which avoid bacterial cells to overlap and allow a more realistic colony growth, and the conjugation process. The state variable update is asynchronous. The order of execution of this process is shuffled to avoid any bias due to a purely sequential execution of model rule base, see Figure 5. The conjugation process is modeled in three different ways with respect to the time when conjugation event is most prone to happen, and the results are compared. Thus, the conjugation is defined by two variables: the value of intrinsic conjugation rate ($\gamma_{0}$), which determines how many transfers should be performed by a single bacterial cell, and the cell cycle point, which defines the time when the conjugative events are likely to occur.

The model input and initialization requires the parameters shown in Table 4. The *costT*4*SS* is the total cost of pili expression. The cost applied for a single pilus expression is *costT*4*SS*/*param*(*maxpili*). The *param*(*maxpili*) is actually a constant having the value of 5 for *Escherichia coli*. The *cellCycle* parameter indicates two things: the type of modeling rule and its parameter. A value of −1 set the model to conjugate as soon an infected cell finds a susceptible one. Setting the parameter to 0 will randomize the conjugation time between $t_{0}$ and *G*. Finally, using a value >0 indicates the specific point in the cell cycle for conjugation. A polynomial equation is fitted to the experimental data where the dependent variable represents the conjugation rate *T*/(*T* + *R*). Setting *isConjugative* flag to false creates a simulation where the transconjugant cells are sterile; in other words, they are unable to conjugate. The *equation* is used only for comparing the quality of simulation output.

**Table 4:** The complete list of model initialization parameters

Parameter	Unit	Description
G	Minutes	Average doubling time for plasmid‐free cells
cellCycle	% of G	The percentage of cellular cycle for conjugation
costConjugation	% of G	The penalization due to a conjugative event
costT4SS	% of G	The Pilus expression cost
$\gamma_{0}$	Conjugations/cell	Upper limit for conjugations performed by an agent
isConjugative	True|false	Defines a conjugative or a mobilizable plasmid
isRepressed	True|false	The T4SS expression state for the plasmid
$N_{0}$	Cells/ml	Initial population expressed in cells/ml
donorRatio	% of $N_{0}$	The initial density of donor cells (*D*)
Equation	N/A	An equation for experimental data

#### 5.1.4 Design concepts

Basic Principles: Three models differing in the way the conjugation rule is implemented and their results compared to the available experimental data. The best strategy can be used to build models which could serve as a predictive tool for synthetic biology and to explore some aspects which are hard to observe directly in experimental studies of plasmid spread. The key points of this model lies on the idea of the existence of a local or intrinsic conjugation rate, which has been termed $\gamma_{0}$. This intrinsic rate stands for the number of plasmid transfer events, or conjugations on a cell life‐cycle basis. In addition, the global infective wave speed depends directly from the specific point in the bacterial cell cycle when conjugative event is triggered.
Emergence: The model intends to find out what will be the global outcome arising as function of local rules defining the evolution of the bacterial cells and their interaction with adjacent neighbors. With this objective, the model incorporates the most significant aspects of the spatial structure and the behavior of the cellular processes that are related to the conjugation. Specifically, the values of the generation time of donor and transconjugant cells are one of the emergent properties depending from the metabolic penalizations applied both for conjugation event and for the expression of T4SS genes.
Adaptation: All agents adapt their growth according to the local availability of nutrient and space.
Fitness: It is considered implicitly to the extent that plasmid‐free individuals will present a better adaptation in terms of growth rate than plasmid bearing cells.
Prediction: The model is intended to provide prediction regarding the range of possible values for the number of plasmid transfer events per cell cycle and the cell‐cycle point when conjugative transfer is most likely to happen.
Sensing: All process defined over the agents implicitly sense the local environment and the close neighborhood for their decisions.
Interaction: Bacterial cells interact with their nearby individuals for nutrient access, cellular division, mate pair formation, and plasmid transfer.
Stochasticity: Stochasticity is introduced at individual level for all cellular process sampling a normal deviate and fitting the value to corresponding process.
Collectives: No collectives are taken into account in this model.
Observation: The output target variables will be saved at intervals of 1 min of simulated time.

#### 5.1.5 Initialization

The simulation model is initialized with a population of plasmid‐free (*R*) and plasmid‐bearing (*D*) cells according to input parameters. The agents are placed randomly within a circular surface centered over the lattice central position. The radius of circle where agents are placed is calculated as function of $N_{0}$ in order to be consistent to the desired initial cell density (Zhong, Droesch, Fox, Top, & Krone, 2012). The simulation environment is also initialized with a number of nutrient particles in order to support the half of the estimated number of cellular divisions, and the rationale behind it is to capture the intercellular competition for nutrient access.

### 5.2 Model analysis

**Figure 6:**
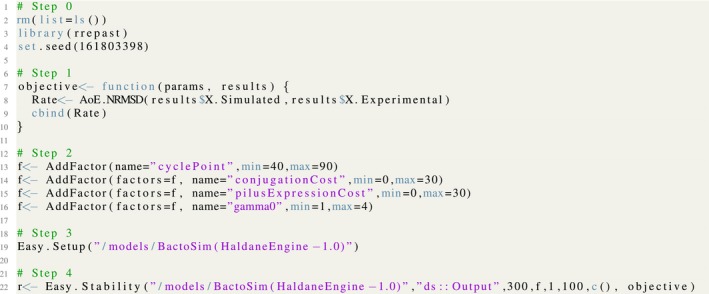
The listing for stability of model output method using the *Easy*.*Stability* function from R/Repast

The objective of stability analysis is to find the minimum number of experimental setup replications required for achieving reliable results. Thereby, the model output response is evaluated for an increasing number of repetitions allowing the evaluation of the convergence for output variance of simulation outputs. The complete listing for carrying out the stability check for the BactoSIM model is shown in Figure 6. As can be observed, the complete implementation of model analysis encompasses five steps. These steps are conserved for all high‐level functions available in R/Repast package. The step 0 clean all existing R objects, loads the R/repast package, and set the random seed for the analysis. The step 1 is the definition of the objective function which can be any user‐provided function following the R/Repast API specification. It is not strictly necessary for the Easy.Stability as the coefficient of variation is calculated for the model output variables. In this example, the objective function is basically the comparison of simulated data and experimental data using the normalized root‐mean‐square error API call *AoE.NRMSD*. The step 2 adds the model input factors for which the importance on the model output will be assessed and their biologically relevant range of variation. It is necessary to add at least one parameter which will be varied, while all other model parameters are kept fixed using the default value or with a value previously set using the R/Repast API *SetSimulationParameter*. The purpose of step 3 is to configure automatically the Repast model with the integration broker and for initializing the integration directory. Finally, the step 4 is where the analysis method is invoked; all analysis methods will return a list holding three objects, namely the *experiment*, the *object*, and the *charts*. The experiment contains simulation parameters and results, the object is method specific, and finally, the charts are pregenerated graphs for the method results.^3^

**Figure 7:**
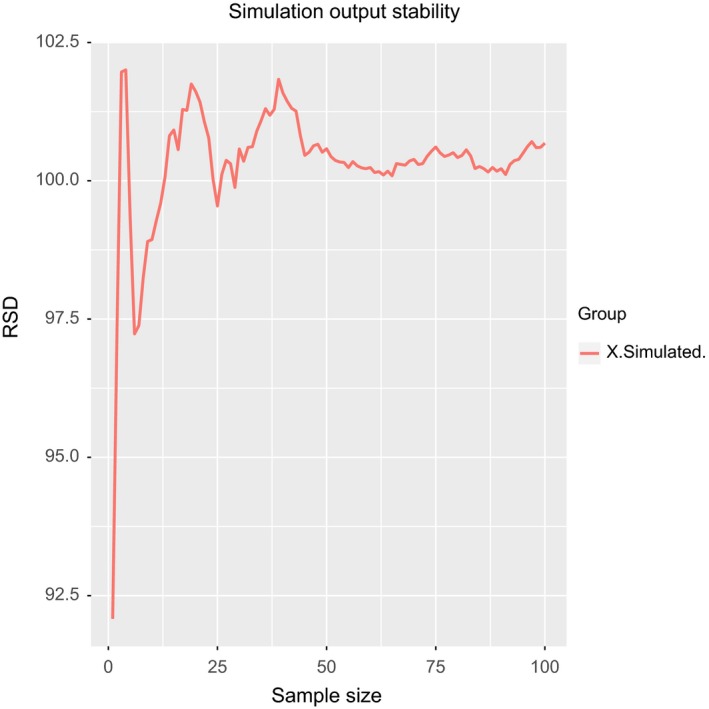
The stability of model output. It is possible to observe how, insofar that the number of replications of the experimental setup increases, the value of the coefficient of variation converges to a common value

The method will generate automatically one chart for each model output.^4^ One of the output chart of model is shown in Figure 7 for the variable named *X.Simulated*. As can be observed, the coefficient of variation of these variable decreases as the sample size increases. The variation starts to become acceptable with a sample size of 25, and approximately with sample size of 50, we can see that coefficient of variation become stable. Therefore, we can feel relatively confident with or model results with a number of replications >25. Of course, it is important to take into account the computation cost of our model in order to select a value for the number of repetitions.

## 6 Example 2: Predator–Prey

### 6.1 Model description

#### 6.1.1 Purpose

The purpose of Predator–Prey model presented here is to provide an alternative individual based‐model implementation for the classic ODE model describing the association between two species. The model will be used to show the application of Morris method for ranking the most important parameters.

#### 6.1.2 Entities, state variables, and scales

The model comprises three entities or agent types, the wolves, the sheep individuals, and the grass. These agents evolve in a computational domain of a 50 × 50 units with periodic boundaries, representing a large portion of space. The agents are positioned in a continuous bidimensional space and are free to move. On the other hand, the grass agent is placed in a discrete grid.

#### 6.1.3 Process overview and scheduling

The agents are defined by the execution of a set of processes depicting the agent movement and search of food source, the consumption of food, the process incrementing the agent reserves, the reproduction, and finally, the death process driven by predation or starvation. The fundamental idea behind the model formulation is that both predator and prey individuals incrementing their “energy” levels by predation or by consuming the available grass, respectively. Both agent types search for their food in the current patch where they are placed. The agents move a unit of space at time selecting randomly the heading.

The individual‐based version of this model is a spatially explicit representation and have a few parameters more but is still very succinct. The list of model parameters are shown in Table 5.

**Table 5:** The input parameter collection for the Repast implementation of Predator–Prey model

Input parameter	Description
initialnumberofwolves	The initial population of predators
initialnumberofsheep	The initial population of preys
wolfgainfromfood	The rate of predator energy is incremented every time a prey is consumed
wolfreproduce	The reproduction rate of predator individual
sheepgainfromfood	The prey rate energy increment for grazing grass
sheepreproduce	The reproduction rate of prey individual
grassregrowthtime	The amount of time required for grass be available again once consumed by a prey

The original formulation of Lotka‐Volterra consists in a system of two differential equations with four parameters, namely the predator and the prey growth rate, the effect of predator on the prey growth, and finally, the effect of prey on the predator growth as can be seen in Equation (9).

(9) $$\begin{array}{cc} \frac{dx}{dt} & =c_{1}x-c_{3}xy,\\ \frac{dy}{dt} & =c_{2}y+c_{4}xy. \end{array}$$

There is a conceptual correspondence between the predator $c_{2}$ and prey $c_{1}$ growth rates with the model parameters *wolfreproduce* and *sheepreproduce* as well as between the parameter *wolfgainfromfood* and the constant $c_{4}$.

### 6.2 Model analysis

**Figure 8:**
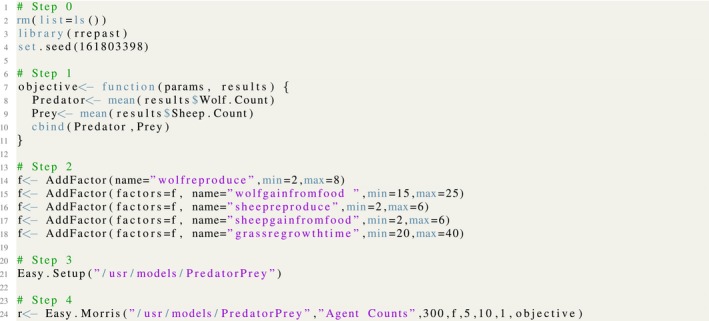
The listing for Morris screening method using the *Easy*.*Morris* function from R/Repast

The implementation code for the Morris screening exercise is shown in Figure 8 and, as has been mentioned in the previous example, we have the same sequence of steps, starting with the library loading and the selection of the random seed. Subsequently, we define the objective function, which in this case is a very simple one consisting in the arithmetic average of the population sizes of sheep individuals and wolves. The next step is the selection of model input factors for the screening method and providing the range of variation for each them. Then, the step 3 shows the call to the *Easy.Setup* function which initializes the Repast Model with the R/Repast integration code. Finally, the function *Easy.Morris* is called and the results are stored in the variable *r*. The example uses five levels with 10 sampling points for Morris method. The results consist of an R list holding the experiment carried out, the Morris object, and a list with charts generated by the experiment.^5^

**Figure 9:**
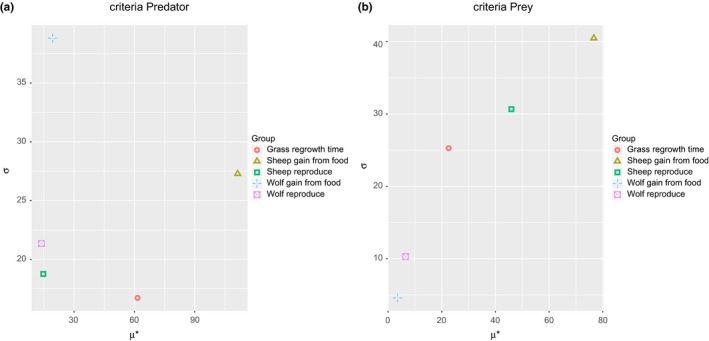
Results of Morris screening method for predator–prey model. The graph shows the $\mu^{*}$ and σ sensitivity measures for Predator (a) and Prey (b) model outputs

The Figure 9 presents the $\mu^{*}$ versus σ chart for both predator and prey average population sizes. At a first glance, the most important input factor for both predator and prey populations is the *sheepgainfromfood*. The second most significant for the predator output is *grassregrowthtime*. The other parameters are not very significant for the average of predator individuals. It is also interesting to note that *wolfgainfromfood* has very high value of σ which could indicate that the parameter significance strongly depends on the values of other parameters. On the other hand, it could mean that the number of sampling points or replications should be increased. The prey output presents three important parameters, which in order of importance are the *sheepgainfromfood*, the *sheepreproduce*, and *grassregrowthtime*. These input parameters also have a high σ values which possibly indicate some nonlinear effects or that the values of these input factors are influencing each other. These results can be explained by the dependence of wolf population on the availability of prey. The common observed pattern in that kind of model is the population of predators lagging in phase behind the prey population.

**Figure 10:**
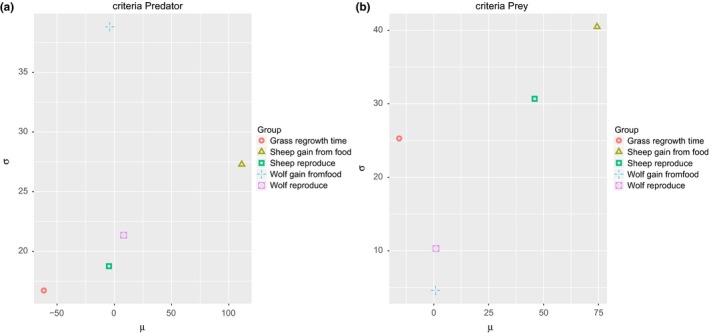
Results of Morris screening method for predator–prey model. The graph shows the μ and σ sensitivity measures for Predator (a) and Prey (b) model outputs

The chart of μ versus σ for model output is shown in Figure 10. It seems to provide very similar results, and the only significant difference is the contribution of *grassregrowthtime*. The input parameter was considered important by μ versus σ, but here, it has a negative value. In order to interpret this sensitivity measure, we must recall that $\mu^{*}$ takes the absolute values of elementary effects. Therefore, the elementary effects of *grassregrowthtime* possibly has effect of opposite sings depending on the values of that input parameter.

**Figure 11:**
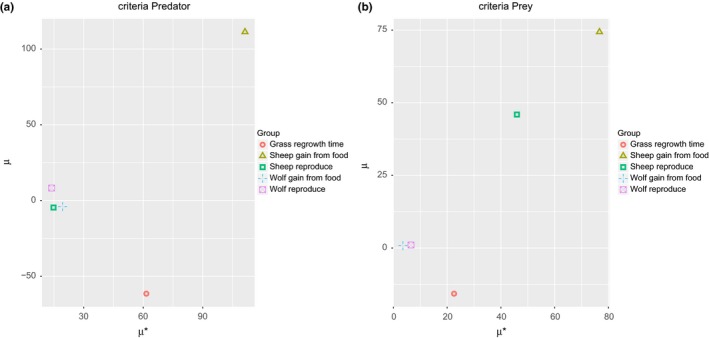
Results of Morris screening method for Predator‐Prey model. The graph shows the $\mu^{*}$ and μ sensitivity measures for Predator (a) and Prey (b) model outputs

Finally, we have the Figure 11 showing the chart of $\mu^{*}$ versus μ, where the value of both measures can be observed together allowing the appreciation of the differences of both, which possibly indicates that the input factors present effects with different signs which, in other words, means nonlinearity in the model behavior.

## 7 Example 3: T4SS Common Pool

### 7.1 Model description

#### 7.1.1 Purpose

The objective of this model is to explore the conditions where two plasmids can coexist in a population competing for a common resource required for their horizontal transfer. The common resource is the set of genes required for conjugation because one of the two plasmid genes has lost these genes.

#### 7.1.2 Entities, state variables, and scales

The model uses two entity types, namely the agents representing the bacterial cells and a *ValueLayer*, which is a Repast specific structure, for holding the nutrient available for the bacterial growth. The agents interact and grow in a computational domain of 100 × 100 μm squared lattice with periodic boundaries representing a total real surface of 0.01 mm${}^{2}$. Despite of being a lattice, the bacterial cells are positioned and allowed to move in a continuous space system. The agents are also allowed to overlap to each other. Explicitly, the agents are defined by the five state variables: (1) heading, (2) mass, (3) division mass, (4) plasmid *P1* infection state, and (5) plasmid *P1* infection state. The current position of every bacterial cell in the coordinate system is available implicitly through a Repast API call.

### 7.2 Process overview and scheduling

Every bacterial cell in this model is abstracted as the execution of a series of successive processes capturing the basic tenets of bacterial life cycle. These processes are the nutrient uptake, the bacterial cell growth, the division, and the conjugation. The input parameters required for initializing the model are shown in Table 6.

#### 7.2.1 Design concepts

Basic Principles: The plasmid dispersion depends on an intricate balance between metabolic costs associated to horizontal and vertical dispersion strategies. The conjugative proficiency requires the expression of set of transmembrane proteins which are known collectively as type IV secretion systems (T4SS; Lawley, Klimke, Gubbins, & Frost, 2003). The presence of conjugative plasmids and the expression of conjugative machinery are detrimental for the host cell fitness (Rozkov et al., 2004), but there is no consensus on the valid ranges of metabolic costs imposed by the conjugative process. Therefore, in this model the short‐term dynamics of two plasmid system *P1* and *P2* is simulated. The plasmid *P1* is a complete conjugative plasmid containing all genes required for horizontal transfer and the plasmid *P2* is a cheater, which having lost its conjugative genes, depends on the T4SS system from the plasmid *P1*. In other words, the model is used to assess how large should be the cost difference required for the lack of conjugative apparatus become a true competitive advantage making *P2* dominate over *P1*.
Emergence: The colony growth pattern, the population distribution, and the dominance of a plasmid over another on the bacterial population are global properties arising from local properties defining the agent behavior and the interaction constraints.
Adaptation: All agents adapt their growth rate, as well as the conjugation rates, according to the local availability of nutrient.
Fitness: The bacterial cells infected by any plasmid are considered to behave less efficiently than the plasmid‐free cells. The fitness of plasmid‐bearing cells is explicitly specified by the cost input parameters.
Objectives: No objectives are taken into account in this model.
Prediction: The model will provide predictions on the possible ranges of plasmid metabolic cost which are favorable to the cheaters plasmid strategy.
Sensing: The agents representing the virtual bacterial cells sense the environment to the extent that the nutrient availability controls the growth and the conjugation rates.
Interaction: Bacterial cells interact with their nearby individuals for nutrient access, cellular division, mate pair formation, and plasmid transfer.
Stochasticity: Stochasticity is introduced at individual level for all cellular process sampling a normal deviate and fitting the value to corresponding process.
Collectives: No collectives are taken into account in this model.
Observation: The model provides two kind of outputs, one is numeric and contains the total number of bacterial cells which are plasmid free or are infected by the plasmids *P1*,* P2*, or both. These outputs are generated for every time step. The model also has a 2D view of colony growth updated every time tick.

**Table 6:** The input parameter collection for the conjugative plasmid common pool model

Input parameter	Description
doublingTime	The doubling time of plasmid‐free cells
p1P ($P(\gamma_{0})$)	The probability of cell conjugate at least one time
p1Cost	The cost imposed by the plasmid P1 including the metabolic burden required to express the conjugative apparatus
p2Cost	The metabolic cost of plasmid P2

### 7.3 Model analysis

**Figure 12:**
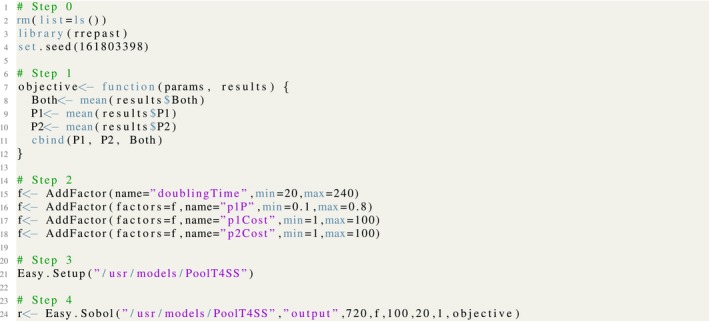
The listing for Sobol GSA variance decomposition method using the *Easy*.*Sobol* function from R/Repast

The global sensitivity analysis using the Sobol variance decomposition method for the T4SS common pool model is shown in Figure 12. We can observe the same sequence of steps which has been previously mentioned. The objective function is defined for the average values of the model outputs named P1, P2, and Both. These variables are, respectively, the bacterial population size infected by the P1 plasmid, infected by the cheater plasmid P2, and finally, the number of individuals infected by both plasmids. The Sobol sensitivity indices will provide the measures of the importance of every input parameter shown in step 2 of Figure 12 with respect to the results returned by the objective function, that is to say, the average population sizes.

**Figure 13:**
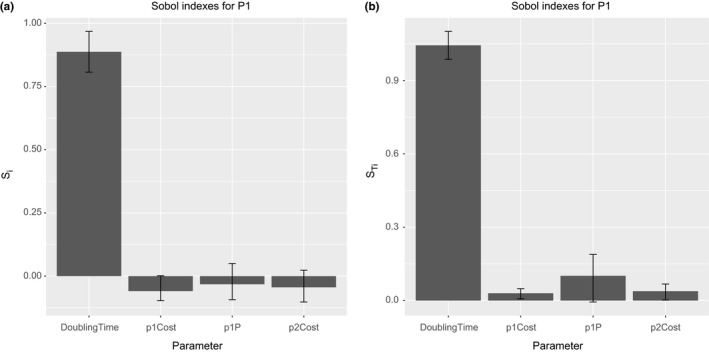
Results of Sobol variance decomposition method for T4SS common pool model. The graph shows sensitivity measures containing the first‐order (a) and total‐order (b) indices for bacterial population infected by P1 plasmid

The Figure 13 shows the first‐ and total‐order indices for the model output P1. That output is the average number of bacterial cells infected just by the plasmid P1. As can be observed, the most important input parameter is the bacterial cell doubling time followed by the probability $P(\gamma_{0})$. This is an expected result as the rule for the conjugative transfer requires the bacterial cells have achieved a value rounding the 70% of cell mass at division. Other interesting aspect to note is the negative values of first‐order indices. Obviously, the sensitivity indices should not be negative. This is a consequence of a small sample size, and to correct the problem, we must increase it. The other important input factors for the plasmid P1 output are, in order of importance, the probability $P(\gamma_{0})$, the cost of plasmid P2, and the cost of plasmid P1, both with similar sensitivity indices.

**Figure 14:**
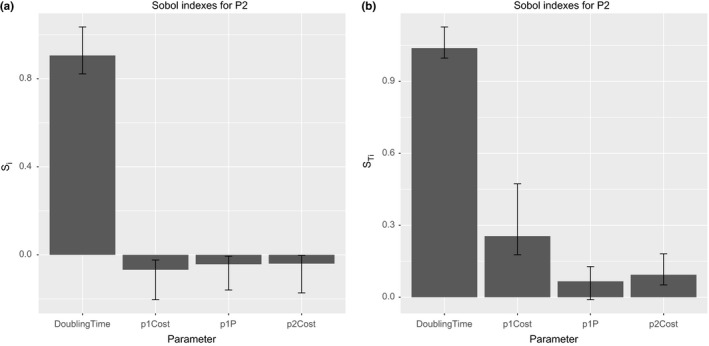
Results of Sobol variance decomposition method for T4SS common pool model. The graph shows sensitivity measures containing the first‐order (a) and total‐order (b) indices for bacterial population infected by P2 plasmid

The first‐ and total‐order indices for the model output showing average population size of plasmid P2 can be seen in Figure 14. It is possible to appreciate again that the sensitivity indices show that the most important factor is the length of cellular cycle. The reason is simple, and can be attributed to the fact that plasmid P2 alone is only transferred vertically and depends on the plasmid P1 for horizontal transmission, being both aspects related to the cell cycle. Following in importance the doubling time, we have the cost of plasmid P1, the cost of plasmid P2, and the probability $P(\gamma_{0})$, being the sensitivity index of P1 cost, noticeably higher than the other two indices. This could be attributed probably because the plasmid P2 requires a significant cost difference in order to outcompete the plasmid P1 which transfer vertically.

**Figure 15:**
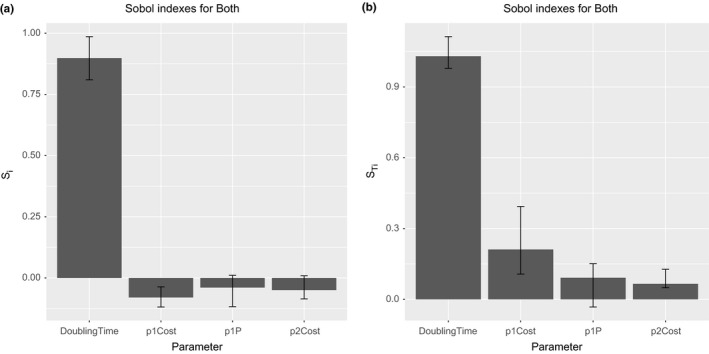
Results of Sobol variance decomposition method for T4SS common pool model. The graph shows sensitivity measures containing the first‐order (a) and total‐order (b) indices for bacterial population infected by both P1 and P2 plasmids

Finally, in the Figure 15, we have the sensitivity indices for the output of model accounting for the average population size of bacterial cells infected by both plasmids. The importance of factors is consistent with the explanations for the previous sensitivity indices. Again, the most important model parameter is the doubling time of bacterial cell followed by the P1 and P2 cost parameters and by the probability $P(\gamma_{0})$.

## 8 Conclusions

The ecological modeling is a complex subject which can be normally perceived as being simpler than it actually is. Specifically, the individual‐based models are subject to many levels of uncertainty, which means that it is hard to get completely fixed the values of model inputs, the model structure, and the outputs. Normally, there is no complete experimental or observational data to construct mechanistic descriptions of individual, and therefore, many assumptions and simplifications must be made in order to implement a model. The same is true regarding the input values, which are particularly critical in the case of the ecology of microorganism, as normally, just very few input parameters are directly observed and the most of them are estimated from whole population experiments. Therefore, it is always important to bearing mind that modeling is an iterative task which must incorporate compulsorily some what‐if analysis of model outputs.

Several methods exist for assessing the uncertainty and for estimating the relative importance of input parameters in the model output. We have provided here and overview on those methods which are based on the variance decomposition because they have a wider application scope and are specifically suitable for their use on individual‐based models. These methods, although conceptually simple, are computationally intensive and can be somewhat hard to apply because the required tools are either unavailable or they do not provide an easy integration pattern. Roughly speaking, the sensitivity analysis methods require the generation of large sample of the parameter space and the model evaluation for each of them which, of course, makes the manual execution an infeasible option.

The in‐silico experimentation is becoming a vital tool for understanding complex phenomena in a way that cannot be performed without modeling. The effective application of computational ecology methods requires a high level of proficiency in many diverse domains of knowledge which sometimes are neither feasible nor practical. Therefore, it is indispensable to have a ready‐to‐use arsenal of reusable computational tools for modeling and analysis. In this study, we have introduced the R/Repast package and showed how it can help modelers to improve the robustness and quality of individual‐based models results using the functionalities inside the package for analyzing systematically the model outputs. The package can save much effort for modelers by providing simple wrappers for complex methods within a simple and consistent API. We hope that these R/Repast functionalities can facilitate enormously the systematic analysis of individual‐based models implemented in Repast.

## Conflict of Interest

None declared.

## Notes

1 Not to be confused with population mean and standard deviation.

2 Also known as relative standard deviation given by CV = σ/μ which provides a normalized version of the standard deviation expressed relatively to the output mean.

3 In the current API version, there is a function for accessing the charts for the *Easy.Stability* method, named Easy.getChart(), please refer to the user manual for the complete syntax.

4 It is possible to limit it passing to the method a subset of model outputs.

5 In order to plot the charts, the user should use a R code for accessing the chat list members. There are three members, namely *mustar*,* musigma*, and *mumu*. In order to get the *mumu* chart for the second objective function output, we must use the R call: *r$charts[2,]$mumu*.
